# A critical cartography of domestic violence policies in Mozambique

**DOI:** 10.1186/s12978-021-01215-7

**Published:** 2021-08-09

**Authors:** Eunice Jethá, Ines Keygnaert, Mohamed Seedat, Joaquim Nhampoca, Mohsin Sidat, Kristien Roelens

**Affiliations:** 1 Community Health Department, Faculty of Medicine of University Eduardo Mondlane, Salvador Allende Avenue Nº 702, Maputo, Mozambique; 2 Ministry of Home Affairs, Department of Attendance of Family and Children Victims of Violence, Maputo, Mozambique; 3 International Centre for Reproductive Health, Department of Public Health and Primary Care, Faculty of Medicine & Health Sciences, Ghent University, Ghent, Belgium; 4 Department of Obstetrics & Gynaecology, Faculty of Medicine & Health Sciences, Ghent University, Ghent University Hospital, Ghent, Belgium; 5 Institute for Social and Health Sciences, University of South Africa, Pretoria, South Africa

**Keywords:** Domestic violence, Policies, Mozambique

## Abstract

**Background:**

Domestic violence (DV) affects millions of people worldwide, especially women impacting their health status and livelihoods. To prevent DV and to improve the quality of victims’ lives, Mozambican governmental and non-governmental entities are making efforts to develop adequate policies and legislation and to improve the accessibility of services for victims of DV.

However, a critical review of whether or not current policies and legislation concerning DV in Mozambique are in agreement with international guidelines has yet to be examined. Therefore, this paper aims to map the Mozambican legislative and policy responses to DV. It also strives to analyse their alignment with international treaties and conventions and with each other.

**Methods:**

Through a critical cartography, documents were selected and their content analysed. Some of these documents were not available online, printed versions were not available on the field and some were not up to date. Therefore, we had to search for them via physical office visits at governmental institutions with a responsibility to deal with DV aspects. These documents were listed and analysed for key content applying a framework inquiring on recommendations of international agencies such as World Health Organization. Subsequently, we compared these policies with international conventions and treaties of which Mozambique is signatory and with each other to identify discrepancies.

**Results:**

Overall, six institutions were visited assuring identification of all available information and policy documents on DV. We identified a total of fifteen national DV documents of which five were on laws, one on policy and nine institutional strategic/action plans. Most of the national DV documents focused on strategies for assistance/care of victims and prevention of DV. Little focus was found on advocacy, monitoring and evaluation.

**Conclusions:**

Mozambique has demonstrated its commitment by signing several international and regional treaties and conventions on DV. Despite this, the lack of consistency in the alignment of international treaties and conventions with national policies and laws is remarkable. However, a gap in the reliable translation of national policies and laws into strategic plans is to be found particularly in relation to naming type, beneficiaries, main strategies and multi-sectorial approach.

## Plain language summary

Domestic violence (DV) which involves physical, sexual and psychological threats, is a Mozambican public health problem that compromises the quality of life of the victims and their families. In extreme cases, such abuse can result in fatalities.

In response to DV, the Mozambican government has adopted international treaties and conventions to design national DV prevention and containment policies and laws.

This article aims to describe how national policies, laws and strategic plans define DV, and align with international treaties and conventions as well as with each other. The analysis indicates that the country’s policies, laws and strategic plans do not meet all recommendations of international guidelines. These include those proposed by the United Nations (UN) and the Pan American Health Organization (PAHO).

While strategic plans propose measures directed at preventing the occurrence of DV and providing victim assistance, they make no reference to monitoring and evaluation, data management and advocacy. Given these mixed findings it is proposed that synergy be obtained among legislation, policy and strategic planning documents and that these documents be revised to incorporate a focused multisectoral approach and monitoring and evaluation.

## Background

### Magnitude of domestic violence

Domestic violence (DV) is a worldwide phenomenon threatening the lives of millions of people. It violates their basic human rights [1, 2]. DV is defined by WHO, as the intentional use of force or power, among members of a particular family or by intimate partners. Additionally such incidents do not exclusively occur in the home [3]. DV comprises physical, psychological, sexual and neglect as the most frequent types of abuse. However, female genital mutilation and other non-sexual coercive behavior, as well as traditional practices considered harmful to women have been recently included in the definition of DV [4–6]. DV affects men, children and older adults of both sexes, nevertheless women are the most affected group, threatening their physical, mental and reproductive health [4, 5, 7]. Tendencies to interchangeably use other terminologies instead of DV is common but most frequently emphasis is put on men as perpetrators [6, 8, 9].

Worldwide one in three women have experienced at some point of their lives either physical or sexual abuse perpetrated by an intimate partner [1]. According to population surveys covering high and low-middle income countries (however not including Mozambique), 10% to over 69% of women report having suffered some form of physical or sexual abuse inflicted by an intimate partner at some point in their lives [8, 10, 11].

According to the WHO in some African countries, 37% of women are physically or sexually assaulted by their intimate partner during their lifetime [12–14].

South Africa is one of the countries with the highest rates of violence by intimate partners in the world. There, violence against women by their intimate partners, represents about 62% of interpersonal violence and the number of women murdered by their intimate partner is five times higher than the per capita global average [15, 16].

In Mozambique a sub-Saharan country with an estimated population of 28 million people, and where regional, cultural and socio-political differences exist, but patriarchy and male dominance are notable throughout the country, shaping gender relations and placing women in vulnerable positions [17–19].

Even though DV may have the same patterns and drivers as other countries in SSA, DV prevalence is relatively low in Mozambique. The Survey on Immunisation, Malaria and HIV/AIDS Indicators in Mozambique (IMASIDA) recently reported that about 24% of women interviewed admitted to being victims of physical (18%), emotional (15%) or sexual violence (3%), supporting to some extent the findings of previous studies [20]. The Mozambican DHS from 2011, showed that although women are most frequently exposed to DV (25%), about 11% of the men reported having experienced an episode of DV, where the female intimate partner was the perpetrator. Approximately 43% of women in Maputo city and 68% in Zambézia province have suffered spousal abuse. These are women who never sought help or told anyone about their DV [19]. Risk factors of violence against men include unemployment and a previous history of being violent against women. It thus appears woman may assume violence as either a defensive or retaliatory act [19, 20].

In Mozambique DV studies are rare, hence the prevalence data remains scarce in contrast to several other countries in SSA [21]. This may be reflective of weak data collection and management system, unresponsive legal structures, plus victims and their relatives’ reluctance to seek help [22, 23].

As in other countries, DV in Mozambique may be driven by a mix of factors including destructive masculinities (originated by the modulation of men personality with aspects considered negative, linking the male power to the authority and placing the woman in a position of submission), socio-economic circumstances, easy access to firearms, and other behavioural factors [23–28].

### Responses to domestic violence

#### A history worldwide

In 1948, the first international instrument, the Declaration of Human Rights, was developed to address aspects related to the integrity of the individual, political and civil rights. In 1979, the Convention on the Elimination of All Forms of Discrimination against Women (CEDAW) was adopted by the United Nations General Assembly, which prohibits all forms of discrimination against women and recommends all UN member states to take action to eliminate discrimination against women by developing appropriate legislative and other measures [29, 30].

Another important instrument, the Beijing Declaration, is the Platform for Action adopted in 1995 which advocated the equality and participation of women in all spheres of life. This Declaration urged all member States to prevent and eliminate violence against women (VAW) using integrated measures, promoting research and providing health care to victims [31].

Many other instruments, protocols and studies have been developed to support gender equality, censure DV and provide guidelines. The Vienna Declaration on Women Rights of 1993, the World Report on Violence and Health in 2002 and the WHO Multi-country Study on Women’s Health and Domestic Violence Against Women in 2005 are some example of these actions [1, 12, 32, 33]. The identification of WHO focal points of violence, and the call for linkages between government ministries are among the many proposals meant to combat and prevent DV [34].

#### Sub-Saharan Africa

Some African countries have committed to enact the recommendations established by international conventions and treaties they have signed. The *Gender and Development Declaration*, signed in 1997 by the Southern African Development Community (SADC), is one example of this commitment. It recommends the establishment of a standing committee of Ministers responsible for gender issues, the adoption of an advisory committee and the establishment of institutional gender focal points and gender units [35]. Another instrument signed in 2004 is *The Solemn Declaration on Gender in Africa* where African member states re-affirmed their commitment to continue, expand and accelerate efforts on gender equality promotion at all levels [36].

The SADC protocol-Declaration on Gender and Development (1997) adopted by all state members, including Mozambique, demonstrates the commitment of African sub-regional bodies to the reinforcement of gender equality. By ratifying these documents, the states made legal commitments to apply diligence for prevention, investigation and criminalization of VAW, as well as to enact laws [32, 35]. The *Protocol to the African Charter on Human and Peoples' Rights for Women (2003)* and the *Cairo Platform* (1994) are other examples of African legal framework on VAW [37, 38].

Being a signatory to the four international treaties and conventions described above, the member states—including Mozambique—committed to integrate their recommendations into the design of national policies ensuring human rights and reducing DV.

#### Mozambique

Before its independence in 1975, the Mozambican political party FRELIMO, was committed to the establishment of gender equality, empowering women through their involvement in the country's political and financial arena. This decision was strengthened by the foundation of the Mozambican Women Organization (OMM) in 1973. Two years later, the implementation of this commitment was at risk by the occurrence of the post-independence civil war that, to a considerable extent, compromised the population’s basic needs such as access to schools and healthcare, and increased women’s vulnerability to DV. In 1990, almost at the end of the civil war, the Constitution of the Republic stressed the need for women's involvement in political and socialeconomic spheres.

Ratification and adoption of several international treaties, conventions and development of national policies, laws and strategies illustrate the governmental effort to establish human rights, specifically for women. Sociocultural factors led to the creation of local organizations at the community level. They are considered functional instances not part of the state’s justice structure. Community courts and groups such as the Mozambican Traditional Healers Organization (AMETRAMO) and the OMM are examples of institutions established to manage DV problems [18].

#### Rationale and specific objectives

Despite the recognised effort of SADC countries including Mozambique to tackle DV, the impact of prevention actions remains sub-optimal. This is because specific socio-cultural and economic factors hamper the implementation of laws and treaties. For example, the involvement of informal justice systems—conflict resolution mechanisms established at the family level or from community and religious leaders—taking decisions is pointed out as one cause of the failure of the DV comprehensive approach [39–41]. However, these mechanisms are in some cases also described as part of the response to DV, especially with regard to referring victims to the formal care system and offering shelters for DV victims [42].

Other studies pointed out this failure was related to the weak content of policies and laws or with their limited implementation [13, 14]. In South Africa, a critical analysis of the DV Act (1998) described some gaps in its content such as lack of the broad definition of DV. It also lacked clarity of who the beneficiaries were, as well as poor integral protection to the victims [43]. Some studies described the questionable allocation of funds for DV prevention and control programs. Additionally, there were gaps in caregiver training for DV victims, and the lack of systematic DV data collection compromising the proper implementation of policies and laws [44, 45]. To date, the impact of efforts made by the government in designing and implementing strategies to combat and prevent DV has not yet achieved the desired results. Therefore, it is crucial to bring an overview of the national legal framework—specifically to policymakers, implementers and researchers. Understanding the contents of policies could persuade such bodies to conduct reforms according to international recommendations and adapted to the local context, thus, this paper aims to provide a framework of Mozambican policies and laws on DV, analyse a selection of identified Mozambican DV related laws, policies and strategic plans. This will firstly determine how these responds to the magnitude and epidemiology of DV in country. Secondly it will reveal how these relate to international instruments and conventions. And thirdly, how these either converge or diverge from each other.

In addition, this study is complementary to a study which describes the conversion of these policies and laws into practices performed in care services for DV victims [46].

## Methods

### Study site

Information analysed in this study was collected in two, locations with high prevalence of DV: Maputo city in the south of the country and in Zambézia in the centre of Mozambique.

This information was obtained from institutions, purposely chosen, with responsibility assigned by the government to deal with gender issues. 

In Maputo City, these institutions are the Ministry of Gender and Social Action, The Ministry of Health and the Ministry of Home Affairs, while in the Zambézia province, these are represented by the provincial directorates which are Ministerial delegations at provincial level. Therefore, it was necessary to meet with professionals who institutionally respond to all gender-related aspects to provide researchers with the desired information. It was also necessary to involve these sites and individuals given the absence of online documents and to ensure all documents would be addressed.

### Data collection

#### Data sources

Mozambican related DV policies, laws and institutional strategic/action plans data were obtained and described. In this specific study, all national policies, laws and institutional strategic/action plans containing in their titles and/or addresses aspects related to DV, family, gender, gender-based violence, intimate partner violence and gender equality, human rights and child protection, approved at central level—the Assembly of the Republic of Mozambique—or institutional level were included in the study. These DV-related documents were referred to by gender focal points of entities holding gender mandates. To complete the listing of documents, electronic searching at all institutional websites under study was conducted from March to November 2017. In addition to national legislation, all treaties and conventions ratified by Mozambique containing the aspects discussed above were also included.

#### Procedures

This process was specifically developed in five steps:

1. Identification of department holding gender mandate as described in the study sites section;
2. Selection of focal points for gender;
3. Searching and selection of DV-related policies and laws referred by the focal points;
4. Electronic searching of other DV-related policies and laws;
5. Description of the content of the laws and policies through a critical cartography.

Reliable data from the policies, laws and institutional strategic/action plans were obtained through institutional focal points for DV and complemented by online sources. Institutional focal points for gender issues are professionals with experience on gender issues, pointed to ensure an integrated approach, through the implementation of national and international policies and programs. Selection of focal points was facilitated by the network of all institutions having a mandate to deal with gender issues where DV is included.

The first gender focal point was identified at the Ministry of Gender Child and Social Action. This institution takes the responsibility of nationally coordinating the integrated approach recommended by the government. Using snowball sampling, other focal points were identified, selected and were included in interviews about the existence of institutional policies, laws, strategic plans and guidelines. Respondents were also queried about national and international documents used as the basis for the design of their mandate.

To understand the purpose of each law, policy or strategic/action plan, a critical cartography was conducted to describe its content. Cartography in this study refers to the identification, compilation and mapping of all national policies, and laws regarding DV. This critical mapping was done taking into account the origin and the type of document analysed as well as its content. This guaranteed a synthesised illustration of all included documents [47].

### Study variables

The variables of this study were established based on the key components recommended by PAHO and UN, in the design of legislative instruments on VAW. These components are the naming style (the title of the document), beneficiaries, main strategies (evidencing the multisectoral approach) and the existence or not of the definition of DV in its content [48–50].

A set of specific groups were created for each component of the framework mentioned above, (see Table 2). Thus, for the component style, naming the analysed documents were sorted into four groups. These are "Violence Against women", “Women”, "Gender equality", "Human Rights" and "Family". The term Child protection was considered a Human Rights issue. In the case of “domestic violence”, although it is a term under study, the only legal document in Mozambique is the law of DV against women, is the reason why the DV was analysed in the group of VAW.

For the component of beneficiaries, four groups were created: "women", "child", "family" and "population in general". Although women—including young girls—and children were also part of the family, they were dealth with in separate groups because some documents analysed were specific to them. Thus avoiding the dispersion of data the "family" group, incorporates not only documents whose beneficiary is the family in general as well as the elderly.

In the main strategies, eight elements have been taken into account: Prevention, Assistance, DV notification, Advocacy, Capacity Building, Monitoring & Evaluation, Protection and Offender Criminalisation. Although the multisectoral approach is also considered a primary strategy, given its peculiarity, it has been described separately. It was taken into consideration in the incorporation of DV definition of the various forms of DV, more specifically: physical, psychological, sexual and economic violence. “Human rights” has also been included since many documents define DV as a violation of women's human rights.

The group of "other", consists of other forms of DV, such as female genital mutilation, incest, premature marriages, trafficking of women and moral violence.

### Data analysis

Documents were selected, verified, screened and listed according to their origin and type in conventions treaties, declarations (international documents) policies, laws, strategic and action plans (national-Mozambican documents). In addition, dates of adoption or signature and approval were taken into consideration (See Table 1).

**Table 1:** Overview of existing policies in Mozambique and international conventions and treaties on DV

*Conventions and treaties*
United nations convention on the elimination of all forms of discrimination against women (1997)
Vienna declaration on human rights (1993)
Declaration of the fourth world conference on women in Beijing (1995)
African charter on human and peoples’ rights (2003)
Solemn declaration of gender equality in Africa (2004)
Cairo platform (1994)
Declaration on gender and development by the southern African development community (2005)
*Mozambican laws and policies*
Laws
Constitution of the republic (2004)
Criminal code (revised in 2014)
Family law (2004)
Law on domestic violence against women (2009)
Legislation on the promotion of protection of the child's rights (2009)
Policies
Gender policy and strategy for its implementation (2006)
*Mozambican action/strategic plans*
Government five-year plan (2015)
National plan of action for the prevention and combat of violence against women (2007)
Health ministry strategic plan (2013)
Police of the Republic of Mozambique strategic plan (2003)
Strategic plan for institutional development of the ministry of the interior (2008)
Program of care for women and children—department of family and children victims of violence (2014)
Intersectoral mechanism of integrated attendance of women victim of violence (2012)
National action plan for the advancement of women 2010–2014 (2014)
National strategy for basic social security (2016)

The analysis of the documents under study were based on the Walt and Gilson model for the analysis of health policies. It employs the triangulation of context in which the policy is developed, its contents as well as its main actors involved in the process of policy design and implementation [48]. In this specific study, although we have used the model described above, we will only perform the analysis of its contents, not the policy context or main actors. In this process of analysis, it is crucial to recognize not all selected documents had the same approach and systematisation of the content. However, they all contain strategies on prevention and control of DV.

To perform content analysis, key components (described above) for the design of legislative instruments on VAW recommended by PAHO and UN were considered.

After this process of systematising all the analysed documents, it was verified to what extent the content of these documents including their titles, addresses the DV problematic. It also investigated whether or not there is alignment not only of national documents but also with international treaties and conventions.

Because we only have one policy in the documents under review, although we recognise the difference between laws and policies, they will be grouped together to facilitate the process of analysis.

## Results

### Current responses to domestic violence

Overall, six institutions were visited to obtain information and retrieved policy, laws and strategic plan documents on DV. In total, twenty-two documents used to address DV were reviewed, of these seven are international (conventions and treaties). We also involved five Mozambican laws, one policy, and nine strategic/action plans. These documents with a common purpose of preventing and combating DV were developed at different levels of attention, (see Table 1).

### Mozambican framework on policies and laws

As shown in Table 1, a total of fifteen national documents addressing DV issues have been identified, including five laws, one policy and nine) strategic plans.

It can be observed in Table 2 that in the design of most national laws, policies and strategic plans, key elements recommended by UN and PAHO have not been incorporated.

**Table 2:** Description of the key elements recommended for the prevention and combat against DV, existing in the twenty-two (22) documents analysed

Key elements	Conventions treaties and declarations (total—3 worldwide; 4 African)	Mozambican laws and policies (6)	Mozambican action/strategic plans (9)
Naming style	Violence against women (2)	Violence against women* (1)	Violence against women (2)
	Women (0)	Women (0)	Women (1)
	Gender equality (2)	Gender equality (1)	Gender equality (0)
	Human rights (2)	Human rights (1)	Human rights (0)
	Family (0)	Family (1)	Family (1)
	Non-specific (1)	Non-specific (2)	Non-specific (5)
Beneficiaries	Women (4)	Women (2)	Women (2)
	Family (3)	Family (2)	Family (5)
	Children (0)	Children (1)	Children (0)
	Population in general (0)	Population in general (1)	Population in general (2)
Main strategies	Prevention (7)	Prevention (3)	Prevention (8)
	Assistance (5)	Assistance (2)	Assistance (9)
	DV case notification (1)	DV case notification (1)	DV case notification (6)
	Advocacy (0)	Advocacy (0)	Advocacy (2)
	Capacity building (1)	Capacity building (1)	Capacity building (4)
	Monitoring & evaluation (0)	Monitoring & evaluation (0)	Monitoring & evaluation (1)
	Protection (0)	Protection (4)	Protection (0)
	Offender criminalization (0)	Offender Criminalization (2)	Offender Criminalization (1)
Multisectoral approach	Yes (6)	Yes (6)	Yes (5)
	No (0)	No (0)	No (4)
	Non-specific (1)	Non-specific (0)	Non-specific (0)
DV definition	Human rights (3)	Human rights (1)	Human rights (1)
	Economic (1)	Economic (2)	Economic (1)
	Physical (1)	Physical (3)	Physical (1)
	Psychological (1)	Psychological (3)	Psychological (1)
	Sexual (1)	Sexual (3)	Sexual (1)
	Other (1)	Other (3)	Other (1)
	No definition (3)	No definition (3)	No definition (7)

*Law on domestic violence against women—although it has the term DV in its name, was considered violence against women in order to standardize data collection

### Key elements

#### Naming style

Regarding the naming style, it was found that in analysed documents, there is a lack of a specific pattern in their title, making this more accentuated in the strategic plans (5). Although for the two types of documents—laws & policies and strategic plans—in some cases, terms such as violence against women (3) and family (2) were used (see Table 2). This lack of specificity was due to the fact these documents was not only intended to address aspects relating to women, gender or DV, but also to address these aspects within their contents.

#### DV definition

According to Table 2, more than half (10) of all national policies, laws and strategic plans do not include the definition of DV in their content. It is noted that this fact is more pronounced in strategic plans (7) regarding laws and policies (3). Of the few strategic plans analysed had the definition of DV, however there is no standard in the use of DV forms in the definition. Whereas in laws and policies, the most frequently described forms of the definition of DV were physical, psychological and sexual.

#### Beneficiaries

Most of DV related national strategic plans analysed were more directed to the family (5), whereas laws and policies had women (2) and family (2) as beneficiaries. The population in general was taken as main beneficiary in only three of the documents described.

#### Main strategies

Neither laws and policies nor strategic plans had included monitoring & evaluation or advocacy as main strategies of action. Prevention (8), assistance (9), DV case notification (6), advocacy (2), capacity building (4), monitoring & evaluation (1) were more described in the strategic plans in relation to the laws and policies. They were more described as protection strategies (4) and offender criminalisation (2).

The multisectoral approach as a strategy to prevent and combat DV was described separately given its relevance and specificity. As can be seen in Table 2, all laws and policies included this strategy in their content while a little less than half the strategic plans did not describe it (4).

### International framework on DV treaties and conventions

#### Key elements

While almost half of international treaties and conventions did not describe the forms of DV, others address it as a violation of human rights (3). The naming style does not follow a pattern. Some analysed documents included in their titles the terms VAW, while others used gender equality or human rights. The most prevalent beneficiaries were the family (3) and then woman (4). The main strategies of these documents were more towards prevention (7), assistance (5), and the multisectoral approach was described in almost all of them (6).

## Discussion

Mozambican framework on policies and lawsThe results described above reveal the Mozambican government has demonstrated its commitment to prevent and combat DV by adopting and signing international treaties and conventions as well as designing and implementing a set of national laws, policies and strategic plans.

The political will to end DV has been instilled by the UN, which has resulted in the involvement of several countries including Mozambique [51].

For example, the Republic of South Africa has propitious conditions for good practices aimed at combating and preventing DV. One of these examples is the existence of strong and comprehensive gender machinery complemented by a wide range of gender-based laws [52]. However, there are some countries that have difficulties in showing this political will, thus compromising the quality of life of DV victims especially women, as is the case in Algeria [53].

### Key elements

The description of national responses to DV will be made in two main components, which are the title of the document (Naming style) and its content (including the definition of the various forms of DV, beneficiaries, the main strategies and the multisectoral approach separately).

Regarding the terminology used in the analised document titles, there is a notable lack of specific pattern in their titles although they describe in their content some fundamental elements, as is the case of indicating the family as its object. Besides that, and considering that the various forms of DV never occur as isolated events, this review illustrates a lack not only of the definition of DV forms, but also of its description in national documents. Most of the national documents described are more focused upon prevention, assistance, and case notification. Little or nothing has been mentioned about advocacy, monitoring & evaluation, offender criminalization and victim protection. Additionally, it was discovered via analysis that almost all available documents, have a multisectoral component in their content, however, the responsibility of each sector is not shown [17, 34, 50, 54–57].

The lack of specificity in the terminology used in the titles may on the one hand increase coverage of these policies, laws and strategic plans benefiting other vulnerable groups such as the elderly or men. However, at the same time, can increase the vulnerability of women. As an example of the lack of adequacy of terminology used in titles are the Mozambican law of DV 29/2009 entitled “law on domestic violence practiced against women”. This vagueness could place other vulnerable groups at risk of being potential victims of DV. Additionally, it somehow establishes barriers to the identification of victims and provision of care. The lack of specificity of the titles is related to the fact that some of the documents under analysis are institutional strategic/action plans. Therefore, they also address several other aspects related to the mandate of the institution and not specifically to DV [49, 58, 59].

Adequacy of policy terminology as well as the improvement of its content, specifically in relation to the indication of its main beneficiaries, are factors that should be considered in the design of gender-sensitive policies, its implementation, monitoring, and evaluation. To avoid these constraints, some countries such as Sweden, have adopted neutral policies, applicable to both men and women [13, 49, 50, 58–60]. Although these definitions may differ from region to region, given the variety of socio-cultural factors, this identified weakness could imprudently compromise responses to DV [6, 32, 45, 53, 61].

Regarding main strategies, there are some peculiarities about protection which is more advocated in policies and laws while capacity building and DV case notification are more recognised in institutional strategic plans. It can be said that in general, the two types of national documents are converging although there is some specificity in each of them. Is evident the lack of description of advocacy strategies as well as monitoring and evaluation in both types of documents.

Our context, which is similar to those of other countries, the DV approach cannot be limited to only some prevention strategies, taking into account the complexity of this phenomenon. Although assistance and primary prevention strategies were addressed in policies, laws and strategic plan development, there is a need to strengthen advocacy and monitoring & evaluation.

Without advocacy, the process of disseminating the magnitude of the problem, its determinants, and consequences may be compromised, further hindering the reform of relevant policies and laws [62]. At the same time, monitoring and evaluation of interventions and long-term monitoring of policies are crucial, including laws and strategic plans to ensure program planning and implementation supported by evidence [55].

In addition to the elements discussed above, it is recommended for the successful design and implementation of policies on DV that they advocate main strategies including the multisectoral approach [54, 63, 64]. The lack of multisectoral involvement can contribute to the perpetuation of instances of violence, not only by weakening social pressure but also by increasing the vulnerability of victims, given the impunity of offenders, and hence increasing cases of revictimisation. To ensure the effectiveness of these responses there is an urgent need to involve several sectors.

In contrast, in several countries, most national documents reviewed, took into account this recommendation, describing the involvement of assorted institutions in the implementation of strategies aimed at reducing DV. However, the responsibility of each sector is not shown [17, 34, 50, 54–57]

Considering that policies and laws are instruments created at a more central level and serve as guiding instruments for the design of strategic institutional plans, some similarities between these two groups regarding the lack of definition of the various forms of DV were found. Thus, the lack of standardisation of the terms in their titles, even though both recognise women and family as the main beneficiaries. The two groups emphasise the multisectoral approach and are more focused on prevention and assistance strategies.

## Alignment with international treaties and conventions

There is a lack of clear alignment among the international treaties and conventions with the national documents analysed regarding the terms in their titles. However, international treaties and conventions consider DV as a violation of human rights. A major challenge for researchers and policymakers is the lack of uniformity of terms. This makes it difficult to effectively compare legislative documents and instruments which measure DV occurrences in a variety of regions or countries [33, 48].

There is a notable convergence of these two groups of documents analysed with regard to the involvement of different sectors and main strategies contained to respond to DV events. The prevention, DV case notification and assistance were the primary issues described in neglecting advocacy, capacity building, protection and monitoring & evaluation. Several countries acknowledge the importance of data collection and management, taking into account its specificity contributing to the design and monitoring of prevention efforts. The design of effective policies, laws and strategic plans combined with reform are possible with the availability professionals and institutional training [14, 39].

The limitation of this study was related to the search for the documents under analysis, given the lack of physical availability in the institutions under study as well as on their web pages. Although an exhaustive search has been made and many documents have been found, we can hypothesize that we have excluded some of them. In addition the lack of specificity in the terminology of the titles of the documents under analysis, made it difficult to carry out the initial screening, and to overcome this difficulty it was necessary to analyse the content of a larger number than the documents included in the study.

## Conclusion

Mozambique has demonstrated its commitment by signing numerous international and regional treaties and conventions. Despite the efforts made, the Mozambican DV-related policies and laws do not incorporate the key elements recommended for its design. It is noteworthy in most of the documents analyze, and more precisely, in the strategic/action plans, there is a lack of specificity in their titles, although their content addresses aspects related to DV.

It was found that less than half of the policies and laws do not have the definition of the various forms of DV. Even though they have considered strategies of prevention and DV combat, these are more directed towards prevention and assistance of the victims. Therefore, they neglect advocacy, monitoring and evaluation as well as the criminalisation of offenders.

However, there is a certain alignment of national policies, international treaties and conventions regarding naming style, beneficiaries, main strategies and the multisectoral approach. Although there is a national commitment, it is crucial to carry out policy reforms to ensure not only the clarity of the implementers in relation to its purposes, but also the broader coverage in terms of the care provided to victims, taking into account the multisectoral approach improving quality of life for victims and their families.

## Data Availability

The datasets used and/or analysed during this study are available from the corresponding authors on reasonable request.

## Abbreviations

AMETRAMO: Mozambican Traditional Healers Organization
CEDAW: Convention on the Elimination of All Forms of Discrimination against Women
DHS: Demographic Health Survey
DV: Domestic Violence
OMM: Mozambican Women Organization
SADC: Southern African Development Community
SSA: Sub-Saharan Africa
VAW: Violence Against Women
WHO: World Health Organizations

## References

[1] Ruiz-Pérez I, Rodriguez-Madrid N, Plazaola-Castaño J (2013). Inhibiting and facilitating factors to end a violent relationship: patterns of behavior among women in Spain. Violence Vict.

[2] Babu BV, Kar SK (2010). Domestic violence in Eastern India: factors associated with victimization and perpetration. Public Health.

[3] Krug EG, Dahlberg LL, Mercy JA, Zwi AB, Lozano R (2002). World report on violence and health. J Med Liban.

[4] Creighton S, Gill A. 'Harmful' traditional practices: interventions to address gendered forms of violence against women and girls. Abstract 0980 presented at IP Safety. London, United Kingdom. 2010;16(1):A276.

[5] Van Veen S, Verkade A, Ukwuagu C, Muriithi M. Breaking a culture of silence: social norms that perpetuate violence against women and girls in Nigeria. OXFAM. Nigeria. 2018; p. 18. https://oxfamilibrary.openrepository.com/bitstream/handle/10546/620458/rr-breaking-culture-silence-enough-campaign-nigeria-280218-en.pdf. Accessed 15 Nov 2018.

[6] Beninger C (2014). The effectiveness of legislative reform in combating domestic violence: a comparative analysis of laws in Ghana, Namibia and South Africa. Neth Q Hum Rights.

[7] Early Intervention Foundation. Early intervention in domestic violence and abuse. Guy J, Feinstein L and Griffiths A. 2014; p. 103.

[8] Heise L, Ellsberg M. Ending violence against women. Baltimore: Population Information Program. 1999;L(11):45.

[9] Izmirli GO, Sonmez Y, Sezik M (2014). Prediction of domestic violence against married women in southwestern Turkey. Int J Gynecol Obstet.

[10] World Health Organization (1997). Violence against women: a priority health issue.

[11] Ehrenberg L, Lawoko-Olwe W, Loum B, Oketayot K, Akot M, Kiyembe C (2014). Inquiry about domestic violence against women in healthcare Uganda: do practitioner attitudes, role conflicts, efficacy, safety concerns and support networks play a role?. Psychology.

[12] World Health Organization. Global and regional estimates of violence against women: prevalence and health effects of intimate partner violence and non-partner sexual violence. Geneva, Switzerland: 2013; p. 57; ISBN: 978 92 4 156462 5. https://apps.who.int/iris/bitstream/handle/10665/85239/9789241564625_eng.pdf. Accessed 20 Jan 2016.

[13] Horváth E, Zukani M, Eppel D, Kays M, Konare A, Park YS, et al. Gender-based violence laws in Sub-Saharan Africa. 2007; p. 87. Retrieved from https://reproductiverights.org/sites/default/files/documents/GBV_Laws_in_Sub_Saharan_Africa.pdf.

[14] World Health Organization. Global status report on violence prevention. Geneva: World Health Organization. 2014; p. 292. ISBN: 978 92 4 156479 3.

[15] Norman R, Bradshaw D, Schneider M, Jewkes R, Mathews S, Abrahams N, et al., Estimating the burden of disease attributable to interpersonal violence in South Africa in 2000. S Afr Med J. 2005;97(3):653–656 17957838

[16] Gass JD, Stein DJ, Williams DR, Seedat S (2010). Intimate partner violence, health behaviours, and chronic physical illness among South African women. S Afr Med J.

[17] República de Moçambique. Plano Nacional de Acção para Prevenção e Combate à Violência contra a Mulher. 2008; p. 28. Retrieved from http://www.wlsa.org.mz/wp-content/uploads/2014/11/PlanoNacionalViolencia2008.pdf. Accessed 30 June 2017.

[18] Arthur MJ, Mejia M. Instâncias locais de resolução de conflitos e o reforço dos papéis de género. A resolução de casos de violência doméstica. Outras Vozes. 2006; p. 12. Retrieved from http://www.wlsa.org.mz/wp-content/uploads/2014/11/Instancias-locais-de-resolucao-de-conflitos-2006.pdf.

[19] Instituto Nacional de Estatítica, Ministério da Saúde. Moçambique: Inquérito Demográfico e de Sáude. Maputo, Moçambique. 2013; p. 430. Retrieved from https://dhsprogram.com/pubs/pdf/FR266/FR266.pdf.

[20] Ministério da Saúde, Instituto Nacional de Estatística, ICF International. Inquérito de Indicadores de Imunização, Malária e HIV/SIDA em Moçambique, Relatório final. Maputo, Moçambique. 2018; p. 453. Retrieved from https://dhsprogram.com/pubs/pdf/AIS12/AIS12.pdf.

[21] Graham-Kevan N, Zacarias AE, Soares JJ (2012). Investigating violence and control dyadically in a help-seeking sample from Mozambique. Sci World J.

[22] Bibi S, Ashfaq S, Shaikh F, Qureshi PMA (2014). Prevalence, instigating factors and help seeking behavior of physical domestic violence among married women of Hyderabad, Sindh. Pak J Med Sci.

[23] Slegh H (2010). Gender-based violence and women’s search for care in Mozambique. Med Antropol.

[24] Heise L. What works to prevent partner violence? An evidence overview. 2011;1-127. ISBN: 978 0 902657 85 2.

[25] José ZB (2016). From cultural practices to violence against women in Mozambique. UEPG Ci Soc Apl Ponta Grossa.

[26] McCloskey LA, Boonzaier F, Steinbrenner SY, Hunter T (2016). Determinants of intimate partner violence in sub-Saharan Africa: a review of prevention and intervention programs. Springer.

[27] El Feki S, Heilman B, Barker G. Understanding masculinities: results from the international men and gender equality survey (IMAGES)—Middle East and North Africa: executive summary. United Nations Women, Promundo. Cairo and Washington, DC. 2017; p. 233; ISBN: 978-03-3519-460-5.

[28] Zacarias AE, Macassa G, Svanström L, Soares JJ, Antai D (2012). Intimate partner violence against women in Maputo city, Mozambique. BMC Int Health Hum Rights.

[29] African Union. Gender policy. 2009; p. 37. Retrieved from https://www.un.org/en/africa/osaa/pdf/au/gender_policy_2009.pdf.

[30] United Nations (2003). The convention on the elimination of all forms of discrimination against women and its optional protocol handbook for parliamentarians.

[31] United Nations. Beijing declaration and platform for action: Beijing+5 political declaration and outcome. Fourth World Conference on Women. 1995;21(4):277. 10.1017/CBO9781107415324.004.

[32] Beninger C (2013). Combating sexual violence in schools in sub-Saharan Africa: legal strategies under regional and international human rights law. AHRLJ.

[33] Garcia-Moreno C, Jansen H, Watts C, Ellsberg M, Heise L (2005). WHO multi-country study on women’s health and domestic initial results on prevalence.

[34] Schopper D, Lormand J, Waxweiler R. Developing policies to prevent injuries and violence: guidelines for policy-makers and planners. World Health Organization; 2006. p. 1–85; ISBN: 92 4 159350 4.

[35] Southern African Development Communities. Declaration on gender development; 1997. p. 5. Retrieved from https://www.sadc.int/files/7613/5292/8380/Declaration_on_Gender__Development_1997.pdf.

[36] African Union. Solemn declaration of gender equality in Africa. Addis Ababa, Ethiopia. Sci Technol 2004;6:1–5.

[37] United Nations Population Fund. Program of action adopted at the international conference on population and development. J Chem Inf Model. 2004;53(9):177. 10.1017/CBO9781107415324.004.

[38] African Union. African (Banjul) charter on human and peoples’ rights. Banjul, Ghana. 1987;14:82. 10.1080/03050718.1987.9985943.

[39] Webster K. Preventing violence before it occurs: a framework and background paper to guide the primary prevention of violence against women in Victoria. VicHealth. Victoria, Australia. 2006:72, 2006. Retrieved from https://www.vichealth.vic.gov.au/media-and-resources/publications/preventing-violence-before-it-occurs.

[40] United Nations Development Fund for Women. What's being done about violence against women and girls: mapping Kenya's civil-society organizations.2007;24. Retrieved from https://www.endvawnow.org/uploads/browser/files/mapping_report_kenya_unifem_2009.pdf.

[41] Odero M, Hatcher AM, Bryant C, Onono M, Romito P, Bukusi EA, Turan JM. Responses to and Resources for Intimate Partner Violence: Qualitative Findings From Women, Men, and Service Providers in Rural Kenya. J Interpers Violence. 2014;29(5): 783-805.ISSN 0886-2605. 10.1177/0886260513505706. 10.1177/0886260513505706 PMC3910289 24255067

[42] Brossoie N, Roberto KA. Community professionals’ response to intimate partner violence against rural older women. J Elder Abuse Negl. 2015;27(0):470–488. 10.1080/08946566.2015.1095664. 10.1080/08946566.2015.1095664 PMC4719151 26421886

[43] Mogale RS, Burns KK, Richter S. Violence against women in South Africa: policy position and recommendations. Violence Against Women, SAGE. 2012;18(5):580–94. 10.1177/1077801212453430; ISSN: 10778012. 10.1177/1077801212453430 22826009

[44] Kruger HB (2004). Addressing domestic violence: to what extent does the law provide effective measures?. J Jurid Sci.

[45] Bravo MMP, Martínez PA, Ruiz IJ (2017). Public policies, nursing role and health programs against gender violence. Comparative study Spain—Brazil. Proc Soc Behav Sci.

[46] Jethá E, Keygnaert I, Martins E, Sidat M, Roelens K. Domestic Violence in Mozambique From policy to practice. BMC Public Health. 2021;21:772. 10.1186/s12889-021-10820-x. 10.1186/s12889-021-10820-x PMC8061038 33888119

[47] Tractenberg L (2013). Applying knowledge cartography techniques and tools to facilitate the process of realist synthesis. Electron J Bus Res Methods.

[48] Walt G, Gilson L (1994). Review article: reforming the health sector in developing countries: the central role of policy analysis. Health Policy Plan.

[49] Solano P, Velzeboer M. Componentes clave en la formulación de leyes y políticas contra la violencia hacia las mujeres (Documento de discusión). Organ. Panam. la Salud Unidad Género y Salud. 2003; p. 25.

[50] Ortiz-Barreda G, Vives-Cases C (2013). Legislation on violence against women: overview of key components. Rev Panam Salud Publ.

[51] Department of Economic and Social Affairs, Division for the Advancement of Women. Handbook for legislations on violence against women. United Nations, New York. 2010; p. 68; ISBN: 9789211302905.

[52] Vetten L. Addressing domestic violence in South Africa: reflections on strategy and practice. United Nations, Division for the Advancement of Women. Vienna, Australia. 2005; p. 1–12.

[53] United Nations. Economic Commission for Africa. African Centre for Gender and Social Development. Violence against women in Africa: a situational analysis. United Nations, Economic Commission for Africa. 2010; p. 1–184. Retrieved from https://projects.iq.harvard.edu/violenceagainstwomen/publications/violence-against-women-africa-situational-analysis.

[54] Ostlin P, Eckermann E, Mishra US, Nkowane M, Wallstam E (2006). Gender and health promotion: a multisectoral policy approach. Health Promot Int.

[55] Day A, Chung D, O’Leary P, Justo D, Moore S, Carson E, Gerace A. Integrated responses to domestic violence: legally mandated intervention programs for male perpetrators. Trends Issues Crime Crim Justice. 2010;(404):8. ISSN: 08178542.

[56] World Health Organization. Preventing intimate partner and sexual violence against women: taking action and generating evidence. Geneva: World Health Organization; 2010. 16:1–102. ISBN: 9789241564007. Retrieved from http://whqlibdoc.who.int/publications/2010/9789.

[57] World Health Organization. Responding to intimate partner violence and sexual violence against women. Geneva: World Health Organization; 2013. p. 1–68. 10.1136/bmj.f3100; ISBN: 9789241548595.

[58] Borwankar R, Diallo R, Somerfelt EA (2008). Gender-based violence in sub-Saharan Africa: a review of demographic and health survey findings and their use in national planning.

[59] Osório C, Cruz e Silva T. Relatório de Pesquisa: entre a denúncia e o silêncio. Análise da aplicação da Lei contra a Violência Doméstica (2009–2015). Women and law in southern Africa. Maputo, Moçambique; 2016. p. 1–286. ISBN: 9789899687189.

[60] República de Moçambique. Orgânica da Assembleia da República. Boletim da República. 2009;I(38):5. Retrieved from http://www.wlsa.org.mz/leisnacionais/.

[61] Tvedten I, Paulo M, Montserrat G. Políticas de Género e Feminização da Pobreza em Moçambique. CHR. Michelsen Institute. 2008:13; ISBN 9788280623379.

[62] República de Moçambique. Política de Género e Estratégias de Implementação. Maputo, Moçambique 2018; p. 1–17. Retrieved from http://forumulher.org.mz/wp-content/uploads/2018/09/politica-de-genero-e-estrategia-implementacao-aprovada-cm-11.09.2018ooo.pdf.

[63] República de Moçambique. Mecanismo Multisectorial De Atendimento Integrado À Mulher Vítima De Violência. 2012;1–77. Retrieved from: http://www.wlsa.org.mz/wp-content/uploads/2014/11/MecanismoMultisectorial.pdf.

[64] World Health Organization. Global plan of action, to strengthen the role of the health system within a national multisectoral response to address interpersonal violence, in particular against women and girls, and against children. Geneva, Switzerland. 2016;1-76. ISBN 9789241511537.

